# Evaluation of Tooth Size in Patients with Congenitally-Missing Teeth

**DOI:** 10.5681/joddd.2013.006

**Published:** 2013-02-21

**Authors:** Barat-Ali Ramazanzadeh, Farzaneh Ahrari, Sara Hajian

**Affiliations:** ^1^Professor of Orthodontics, Dental Research Center, Mashhad University of Medical Sciences, Mashhad, Iran; ^2^Assistant Professor of Orthodontics, Dental Research Center, Mashhad University of Medical Sciences, Mashhad, Iran; ^3^Dentist, Private Practice, Mashhad, Iran

**Keywords:** Hypodontia, microdontia, missing tooth, tooth size, tooth width

## Abstract

**Background and aims:**

Hypodontia is a common developmental abnormality of dentition. This study aimed to determine tooth width in patients affected with mild hypodontia and compare the results with a control group without tooth agenesis.

**Materials and methods:**

The orthodontic records of 25 patients with congenital missing of one or two teeth (hypodontia group), and 25 subjects with full dentition (control group) were selected. The greatest mesiodistal width of each tooth was measured on the study models by a digital caliper. Tooth width measurements were compared between the groups using a student t-test at p < 0.05 of significance.

**Results:**

Patients with hypodontia showed narrower teeth than the control subjects. The differences in tooth size between the two groups were statistically significant for the first and second premolars and first molar in the maxillary right and for the second premolar in the maxillary left quadrants (p < 0.05). In the lower arch, the first and second premolars and also first molar in both sides of hypodontia patients demonstrated significant reduction in tooth size compared to the control group (p < 0.05).

**Conclusion:**

These findings suggest that patients with mild hypodontia have narrower teeth than normal subjects especially in posterior segments, which may have clinical implications during the orthodontic treatment process.

## Introduction

Hypodontia, the congenital absence of one or more (up to six) teeth except third molars, is one of the most common developmental abnormalities of dentition with a prevalence ranging from 3.2% to 13.3% in different populations.^1-6^ The condition is termed oligodontia when the number of congenitally missing teeth (excluding third molars) exceeds six, while anodontia demonstrates complete tooth absence. Genetic, epigenetic and environmental factors contribute to the development of this condition,^7-9^ with genetics showing a predominant role in the etiology of tooth agenesis.^10^ The congenital absence of permanent teeth has been categorized as a handicapping dentofacial anomaly according to World Health Organization,^9^ and it occurs more commonly in females than males.^2,11^ Hypodontia may be a dental manifestation of special syndromes such as ectodermal dysplasia^12^ and cleft lip and/or palate,^13^ or occur as an isolated condition.

Hypodontia is frequently associated with skeletal and dental abnormalities. Previous studies reported changes in craniofacial morphology such as bimaxillary retrusion, decreased maxillary jaw size, mandibular prognathism, and reduced vertical facial dimension in patients affected with hypodontia.^11,14-17^ The association of congenital tooth absence with palatally displaced canines, delayed tooth eruption, infraocclusion of primary molars, enamel hypoplasia, and abnormal shape and size of maxillary lateral incisors has also been demonstrated,^18-24^ frequently requiring orthodontic and restorative interventions to achieve an aesthetic dentition.

There are controversial reports regarding tooth size in patients with congenitally missing teeth. An ample of evidence suggests that patients with hypodontia have smaller crown dimensions than normal subjects in the remainder of the dentition,^8-10^,^16,25-28^ a condition which is termed microdontia. However, Chung et al^2^ concluded that hypodontia was not associated with changes in tooth size, because the overall tooth sizes were similar in the hypodontia and normal groups in both males and female subjects. Yamada et al^29^ found that in subjects with congenital absence of one or two teeth, the remaining teeth were generally larger than the control group. But, when three or more teeth were missing, a significant reduction in tooth size was observed throughout the dentition compared to the controls.^29^ Wisth et al^30^ found that in patients with missing teeth, neither the mesiodistal diameter of the teeth nor the dental arch width were significantly different from a control group without tooth agenesis.

The treatment of patients affected with hypodontia is challenging in most cases, and requires an interdisciplinary approach for proper management. The key question is whether the space should be closed by orthodontic treatment or be opened to place an eventual restoration. Tooth size is not only an important factor to determine crowding or spacing in a jaw but also becomes an important parameter when deciding on space management in the edentulous area.

The aim of this study was to evaluate the mesiodistal crown sizes of remaining dentition in patients affected with hypodontia and to compare them with those of a control group with complete dentition.

## Materials and Methods

Intraoral photographs of patients treated in Department of Orthodontics of Mashhad Dental School, and three private practices were reviewed to select a sample of 25 cases (17 female, 8 male) with agenesis of one or two permanent teeth except third molars (hypodontia group). A control group was also selected comprising of 25 consecutive patients (14 female, 11 male) exhibiting complete dentition. The sample size was calculated according to a previous study,^25^ using the NCSS software (NCSS 2003 and PASS 2002, Number Cruncher Statistical Systems, Kaysville, USA) with α = 0.05, β = 0.12 and power of 88%. The patients ranged in age from 12 to 25 years.

The inclusion criteria for both groups consisted of full eruption of all permanent teeth except third molars, availability of qualified dental casts and pretreatment panoramic radiographs, minimal crowding, and absence of any craniofacial syndromes. Patients who had caries, interproximal restorations and ectopic tooth eruption, as those with previous orthodontic treatment or with history of permanent tooth extraction were excluded from the study. The panoramic radiographs were evaluated to confirm the presence of all permanent teeth (excluding third molars) in the control group and the congenital absence of at least one permanent tooth (excluding third molars) in the hypodontia group.

The mesiodistal dimension of each tooth in both the hypodontia and control groups was determined on pretreatment study models. For this purpose, the maximum distance between the contact points on the two proximal surfaces of the crown was measured parallel to the buccal surface with accuracy of 0.01 mm using a digital caliper (Guanglu, China) modified with special tips for precise tooth size measurement. All measurements were done by one investigator (SH).

Statistical calculation was performed in SPSS software (Statistical Package for Social Sciences, version 11.5, Chicago, IL, USA). A Kolmogorov-Smirnov test confirmed the normal distribution of the data; thus a two-sample t-test was used to detect statistical differences in tooth width measurements between the two groups. The significance level was predetermined at α = 0.05.

To evaluate intraoperator reliability, ten study models were randomly selected from each group and the mesiodistal tooth dimensions were measured again at a one-month interval. The systematic error was determined using a paired-sample t-test, and the casual error was calculated by Dahlberg’s formula,^31^ (Error of method^2^ = ∑d^2^/2n, where d is the difference between the two measurements of a pair and n is the number of double measurements).

## Results

The mean age of patients in the hypodontia group was 16.25 ± 3.59 years and that of the control group was 17.4 ± 3.29. The measurement error was calculated to be in the range of 0.008 to 0.097 mm, and no statistical difference was found between the two measurements (p > 0.05).

Table 1 shows the distribution of agenesis by tooth type in the hypodontia group. All patients were missing one or two teeth. The most common congenitally missing teeth were the upper lateral incisors (52.6%) followed by the lower second premolars (26.3%). In addition, four maxillary second premolars, two maxillary canines and two mandibular lateral incisors were missing in the sample (Table 1). The most common pattern of hypodontia was bilateral missing of upper lateral incisors observed in seven (28%) cases, and then unilateral missing of upper lateral incisor and unilateral missing of lower second premolar were more common which occurred in six (24%) and four (16%) subjects, respectively.

**Table 1:** The distribution of agenesis by tooth type in the hypodontia group

Tooth position	number	%
UR2	10	26.32
UR3	1	2.63
UR5	3	7.89
UL2	10	26.32
UL3	1	2.63
UL5	1	2.63
LR2	1	2.63
LR5	5	13.16
LL2	1	2.63
LL5	5	13.16
Total	38	100

UR: upper right; UL: upper left; LR: lower right; LL: lower left; numbers in ‘tooth position’ column indicate the number of teeth in the Palmer system.

Generally, subjects affected with hypodontia of one or more permanent teeth had smaller tooth widths compared to those in the control group (Table 2). The only exception was found for the upper central incisor where the mesiodistal dimension was slightly greater in the hypodontia than the control group. In the maxillary arch, the greatest difference in the average tooth width between the two groups was 0.45 mm relating to the second premolar tooth (Table 2). The corresponding value in the mandibular arch was 0.47 mm pertaining to the first molar (Table 2). Generally, there were greater differences in tooth width measurements in the posterior than anterior dentition.

**Table 2:** The comparison of tooth width measurements (mm) in the hypodontia and control groups

Tooth position	Hypodentia (Mean ± SD)	Control (Mean ± SD)	Difference	P Value
UR1	0.46 ± 8.03	0.44 ± 7.98	+ 0.05	0.174
UR2	0.65 ± 5.99	0.50 ± 6.21	-0.22	0.173
UR3	0.44 ± 6.96	0.47 ± 7.02	-0.06	0.952
UR4	0.54 ± 6.26	0.40 ± 6.69	-0.43	0.006*
UR5	0.36 ± 6.15	0.38 ± 6.50	-0.35	0.003*
UR6	0.48 ± 9.77	0.42 ± 10.15	-0.38	0.006*
UL1	0.44 ± 8.01	0.39 ± 8.0	+0.01	0.676
UL2	0.6 ± 6.0	0.50 ± 6.28	-0.28	0.067
UL3	0.45 ± 6.96	0.51 ± 7.12	-0.16	0.496
UL4	0.45 ± 6.32	0.36 ± 6.55	-0.23	0.062
UL5	0.45 ± 6.10	0.51 ± 6.55	-0.45	0.004*
UL6	0.51 ± 9.91	0.45 ± 10.06	-0.15	0.388
LR1	0.32 ± 4.97	0.31 ± 4.98	-0.01	0.705
LR2	0.44 ± 5.36	0.43 ± 5.58	-0.22	0.103
LR3	0.33 ± 6.01	0.50 ± 6.19	-0.18	0.401
LR4	0.41 ± 6.40	0.35 ± 6.67	-0.27	0.031*
LR5	0.36 ± 6.49	0.40 ± 6.74	-0.25	0.028*
LR6	0.44 ± 10.05	0.57 ± 10.52	-0.47	0.002*
LL1	0.34 ± 5.0	0.32 ± 5.01	-0.01	0.357
LL2	0.46 ± 5.46	0.40 ± 5.57	-0.11	0.313
LL3	0.34 ± 5.98	0.53 ± 6.23	-0.25	0.091
LL4	0.61 ± 6.34	0.42 ± 6.67	-0.33	0.014*
LL5	0.47 ± 6.51	0.52 ± 6.88	-0.37	0.024*
LL6	0.43 ± 10.16	0.55 ± 10.58	-0.42	0.004*

P value is provided from the two-sample t-test. * indicates statistical significance at p < 0.05. UR: upper right; UL: upper left; LR: lower right; LL: lower left; numbers in ‘tooth position’ column indicate the number of teeth in the Palmer system.

Statistical analysis by the two-sample t-test revealed significant between-group differences in tooth size for the first and second premolars and first molar of the upper right quadrant and also for the second premolar in the maxillary left quadrant (p < 0.05; Table 2). In the lower jaw, statistical differences in tooth size measurements between the two groups were observed in the first and second premolars and also first molar in both the right and left quadrants (Table 2).

## Discussion

In the present study, mesiodistal tooth dimension was compared between patients affected with mild hypodontia and normal subjects. Patients who were missing one or two permanent teeth had narrower teeth compared to the control group throughout the dentition except for the maxillary central incisors. This finding is in agreement with those of previous studies,^8-10,16,25-28^, and indicates an association between hypodontia and smaller tooth size in the remaining dentition. In contrast, Wisth et al^30^ did not find any statistical difference in mesiodistal diameter of the teeth between the hypodontia group and a control group without tooth agenesis. Chung et al^2^ concluded that hypodontia is not associated with reduced tooth size. Yamada et al^29^ reported that tooth size in patients with agenesis of one or two teeth tended to be larger compared to a control group with all 32 permanent teeth present, possibly due to compensatory interactions that affect tooth dimension.^29^

The difference in tooth width between the two groups was more evident in the posterior than anterior segments, reaching a statistical significance for most of the first and second premolars and also first molars. Mirabella et al^10^ reported that in subjects with unilateral or bilateral missing of maxillary lateral incisors, the mesiodistal widths of right and left maxillary first molars were comparable to those of normal individuals, thus they suggested interproximal reduction of maxillary first molars in cases where additional space is required to place an implant-supported restoration in the anterior tooth segment. In contrast to the findings of Mirabella et al,^10^ the present findings indicate a significant reduction in mesiodistal widths of first molars in subjects with congenital missing of one or two permanent teeth. This controversy may be related to the criteria of patient selection, as Mirabella et al^10^ investigated tooth widths in patients with unilateral or bilateral missing of maxillary lateral incisors, but in the present investigation, patients with agenesis of other permanent teeth were also included.

In this study, patients with mild hypodontia did not show a significant reduction in tooth size in the anterior dentition compared to the control group. This finding is in contrast with several studies that reported statistically narrower anterior teeth in patients with missing teeth as compared to normal subjects.^9,26,28^ The lack of statistical significance in the anterior tooth size between the two groups may be related to the criteria of patient selection as only those with one or two missing teeth were included in the study. It has been demonstrated that the degree of decrease in tooth size is related to the number of missing teeth; therefore, as the number of missing teeth increases, the decrease in tooth size of the remaining dentition would be more remarkable.^8,25,32^ Furthermore, agenesis of maxillary lateral incisors occurred bilaterally in most cases, thus the prevalence of peg or small lateral incisors as frequently reported in unilateral cases^10,16,22,33-34^,would be smaller in the sample.

The maximum difference in the average mesiodistal tooth size between the two groups was 0.45 mm in the upper arch and 0.47 mm in the lower arch. These values are similar to the maximum difference of 0.49 mm between the control group and the agenesis group, as reported by Mirabella et al,^10^ but are considerably smaller than those reported by Brook et al^8^ and McKeown et al.^26^ The difference may be related to the small number of agenesis per individual in the present sample, while severe cases were also included in the studies that showed a close relationship between the reduction in tooth size and missing teeth.^8,26,28^ The findings of this study corroborate the results of Brook et al^25^ that in less severe cases of hypodontia, the relationship between the tooth agenesis and the size of the remaining dentition is not strong.

Congenital absence of permanent teeth is a complex condition that requires a multidisciplinary treatment approach. The orthodontist is frequently the first practitioner who detects the problem and decides on closing the space or providing adequate space for future placement of a restoration. Although the decision depends on individualized characteristics such as the type of malocclusion and the need for extraction in the opposite arch as well as to the number of missing teeth, it is important to consider the dental and skeletal anomalies that may be associated with tooth agenesis. For example, the deficient alveolar growth which may be observed in these patients supports the prosthetic replacement of the absent tooth/teeth to improve lip protrusion and patient’s profile. The small tooth size in the remaining dentition as observed in the present study indicates that not only the chance of crowding is low but also space redistribution to provide space for a future restoration may be frequently required in these patients. Furthermore, if canine substitution is going to be performed in cases of congenitally missing lateral incisors, it has been recommended to prevent grinding the canine to a smaller lateral incisor, but restore the canine and also the upper central incisor and the first premolar to obtain wider teeth, thus achieving a more esthetic and functional result.^35-36^ However, either enlargement of the lower anterior teeth or thickening of the upper anterior restorations would be required in this case to counteract the resultant interarch tooth size discrepancy.^35^

It is also important to create sufficient interradicular space when treatment planning includes placing an implant-supported restoration or autotransplantation. Previous authors emphasized that in patients affected with hypodontia, it may be challenging to provide adequate space for implant placement especially in the anterior dentition,^10^ because the whole dentition is small in size and thus the interradicular space would be proportionately less than that of normal subjects. The findings of the present study, however, indicate that interradicular space may not be a great concern in patients affected with mild hypodontia because the difference in tooth size between the two groups was small in most areas.

## Conclusion

Patients affected with mild hypodontia showed smaller mesiodistal crown size compared to the control group in the remaining dentition. The reduced tooth size was more evident in the posterior segments than in the anterior. This should be considered during treatment planning and deciding on treatment mechanics in order to achieve a functional occlusion and an esthetic dentition at the end of the orthodontic treatment.
